# Immune Checkpoint Inhibitor–Associated Tumor Lysis Syndrome: A Real-World Pharmacovigilance Study

**DOI:** 10.3389/fphar.2021.679207

**Published:** 2021-09-23

**Authors:** Li Wang, Xiaolin Li, Bin Zhao, Dan Mei, Jiandong Jiang, Jingli Duan

**Affiliations:** ^1^ Department of Pharmacy, Peking University International Hospital, Beijing, China; ^2^ Department of Pharmacy, Peking Union Medical College Hospital, Peking Union Medical College, Chinese Academy of Medical Sciences, Beijing, China; ^3^ State Key Laboratory of Bioactive Substance and Function of Natural Medicines, Institute of Materia Medica, Peking Union Medical College, Chinese Academy of Medical Sciences, Beijing, China

**Keywords:** immune checkpoint inhibitor, tumor lysis syndrome, cytotoxic T lymphocyte antigen-4, programmed death receptor-1, programmed cell death 1 ligand 1, pharmacovigilance

## Abstract

**Introduction:** Immune checkpoint inhibitors (ICIs) have substantially improved the clinical outcomes of various malignancies. However, the adverse event of tumor lysis syndrome (TLS) has not been included in the National Comprehensive Cancer Network guidelines or drug inserts. In this study, we aimed to establish the relationship between ICI therapies and TLS events using data from a real-world pharmacovigilance database.

**Methods:** The MedDRA terms of TLS and both generic and brand names of ICIs were retrieved from the FDA Adverse Event Reporting System. Four frequentist algorithms were employed to confirm the association between the TLS and the ICI regimens, involving anti-cytotoxic T lymphocyte antigen-4 (anti-CTLA-4), anti–programmed death receptor-1 (PD-1)/programmed cell death 1 ligand 1 (PD-L1), and anti-(CTLA-4 + PD-1). A descriptive and statistical analysis was performed according to the case information.

**Results:** One hundred sixty-four TLS cases, where patients underwent anti-CTLA-4 (*n* = 14), anti-(PD-1)/(PD-L1) (*n* = 113), or anti-(CTLA-4 + PD-1) (*n* = 37) therapies, were collected between the first quarter of 2004 and the fourth quarter of 2020. The most coverage-reporting year, age-group, sex, reporter, region, country, and indication were 2020 (*n* = 62), 60–74 years (*n* = 65), males (*n* = 105), physician (*n* = 66), Asia (*n* = 80), Japan (*n* = 67), and lung and thymus malignancies (*n* = 40), respectively. The median TLS onset time associated with anti-CTLA-4, anti-(PD-1)/(PD-L1), and anti-(CTLA-4 + PD-1) therapies was 6 (IQR: 2–39.5), 9 (IQR: 2–40), and 20 (IQR: 7.5–37.75) days, respectively. Mortality distribution of 71 reported death outcomes among three groups was statistically significant. All four algorithm signal values of anti-(CTLA-4 + PD-1) were higher than those of anti-CTLA-4 and anti-(PD-1)/(PD-L1).

**Conclusion:** Elderly male patients with lung and thymus malignancies are frequently predisposed to TLS. ICI therapies could induce TLS in both solid and hematological malignancies. The rapid onset time and poor outcomes of patients prompt caution from health-care professionals.

## Introduction

By blocking cytotoxic T-lymphocyte antigen-4 (CTLA-4) or targeting the programmed death receptor-1 (PD-1)/programmed cell death 1 ligand 1 (PD-L1) pathway, immune checkpoint inhibitors (ICIs) have transformed the treatment landscape for a range of neoplasms (Hodi et al., 2010; Borghaei et al., 2015; Brahmer et al., 2015; Le et al., 2015; Reck et al., 2016; Rittmeyer et al., 2017). Since their introduction, both mono- and combined ICI regimens have substantially improved outcomes in patients with solid and hematologic malignancies.

Tumor lysis syndrome (TLS) is a life-threatening condition that can occur when massive malignant cells die and break down quickly, releasing large amounts of nucleic acids, phosphate, potassium, and cytokines into the circulatory system either spontaneously or in response to treatment (Cairo et al., 2010; Howard et al., 2011). According to the Cairo–Bishop classification criteria, laboratory TLS requires two or more metabolic disorders such as hyperuricemia, hyperkalemia, hyperphosphatemia, and hypocalcemia to occur during the same 24-h period within 3 days before the initiation of therapy or up to 7 days thereafter. Clinical TLS is defined as the presence of laboratory TLS along with an increased creatinine level, seizures, or cardiac arrhythmia/sudden cardiac death (Cairo and Bishop, 2004).

The Food and Drug Administration (FDA) Adverse Event Reporting System (FAERS) is a spontaneous reporting system (SRS) database that contains adverse event reports, medication error reports, and product quality complains. Post-marketing pharmacovigilance is often used to depict the surveillance activities. Data mining algorithms based on disproportionality analysis could assist experts in predicting adverse events of drugs and therapeutic biologic products.

Various immune-related adverse events can occur during treatment with ICIs according to the tumor type, and they commonly involve the gastrointestinal tract, endocrine glands, skin, and liver (Postow et al., 2018). To the best of our knowledge, there are no peer-reviewed pharmacovigilance studies of ICI-induced TLS to date and their possible relationship is not mentioned in the National Comprehensive Cancer Network guidelines (Thompson et al., 2020) or the package inserts (ASTRAZENECA_UK_LTD; BRISTOL_MYERS_SQUIBBa; BRISTOL_MYERS_SQUIBBb; EMD_SERONO_INC; GENENTECH_INC; MERCK_SHARP_DOHME; REGENERON_PHARMACEUTICALS). Therefore, the aim of the present study was to establish the relationship between ICI regimens and TLS adverse events using data from the FAERS database.

## Methods

### Data Mapping

Institutional review board approval for the present study was not required as the FAERS database is accessible to the public. The Medical Dictionary for Regulatory Activities (MedDRA) is the international medical coding system developed under the auspices of the International Council for Harmonisation of Technical Requirements for Pharmaceuticals for Human Use (ICH). “Tumour lysis syndrome” [20000219] is a standardized MedDRA Query (SMQ), which comprises three preferred terms (PTs) “hemorrhagic tumour necrosis” [10054096], “tumour lysis syndrome” [10045170], and “tumour necrosis” [10054094].

The FDA-approved generic (and brand names) of ICIs are ipilimumab (Yervoy^®^), nivolumab (Opdivo^®^), pembrolizumab (Keytruda^®^), cemiplimab (Libtayo^®^), atezolizumab (Tecentriq^®^), avelumab (Bavencio^®^), and durvalumab (Imfinzi^®^). These ICIs were grouped into “anti-CTLA-4” (ipilimumab only), “anti-(PD-1)/(PD-L1)” (monotherapy of nivolumab, pembrolizumab, cemiplimab, atezolizumab, avelumab, and durvalumab), and “anti-(CTLA-4+PD-1)” (combined therapy of ipilimumab and nivolumab only) in this study. The FDA-approved labels of the seven drugs were downloaded on January 26, 2021, and the latest revised indications were clustered according to the World Health Organization (WHO) classification of tumors.

All four adverse event terms and 14 ICI names were retrieved from the FAERS database. The FAERS allows the output of seven data categories, namely, demographic and administrative, drug information, adverse events, patient outcomes, report source, start and end dates of therapies, and therapeutic indications.

### Signal Detection

Based on disproportionality analysis, the frequentist algorithms, including reporting odds ratio (ROR) and proportional reporting ratio (PRR), and the Bayesian algorithms, comprising Bayesian confidence propagation neural network (BCPNN) and multi-item gamma Poisson shrinker (MGPS), were employed to calculate the association between the use of ICIs and cases of TLS (van Puijenbroek et al., 2002; Hauben et al., 2005; Matsushita et al., 2007). Notably, the corresponding signal score metrics are expressed as confidence interval (CI), chi-squared (χ^2^) test value, information component (IC), the lower limit of the 95% two-sided CI of the IC (IC025), empiric Bayes geometric mean (EBGM), and the lower 90% one-sided CI of EBGM (EB05) (Chen et al., 2020).

Based on the 2 × 2 contingency table, *a* represents reports of the suspected drugs with the adverse drug reaction (ADR) of interest, *b* represents reports of the suspected drugs without the ADR of interest, *c* represents reports of other drugs with the ADR of interest, and *d* represents reports of neither the suspected drugs nor the ADRs of interest. The formulae and criteria of four algorithms for signal detection are given in Table 1 (Hauben, 2003; Hauben et al., 2005).

**TABLE 1:** Formulae and criteria of four algorithms for signal detection.

Algorithm	Formula	Criteria
ROR	$\text{ROR}=\frac{\left(a/c\right)}{\left(b/d\right)}$ ; $95\text{\%CI}=e^{\text{ln}\left(ROR\right)\pm 1.96\sqrt{\left(\frac{1}{a}+\frac{1}{b}+\frac{1}{c}+\frac{1}{d}\right)}}$	95% CI>1, N≥2
PRR	$\text{PRR}=\frac{a/\left(a+b\right)}{c/\left(c+d\right)}$ ; χ^2^ $=\sum^{\text{​}}\frac{{\left(a-E\right)}^{2}}{E}$ , E = $\frac{\left(a+b\right)\left(a+c\right)}{\left(a+b+c+d\right)}$	PRR≥2, χ^2^≥4, N≥3
BCPNN	$\text{IC}=log_{2}\frac{a\left(a+b+c+d\right)}{\left(a+c\right)\left(a+b\right)}$ ; $\text{IC}025=e^{\ln\left(IC\right)-1.96\sqrt{\left(\frac{1}{a}+\frac{1}{b}+\frac{1}{c}+\frac{1}{d}\right)}}$	IC025>0
MGPS	$\text{EBGM}=\frac{\text{a}\left(\text{a}+\text{b}+\text{c}+\text{d}\right)}{\left(a+c\right)\left(a+b\right)}$ ; $\text{EB}05=e^{\ln\left(EBGM\right)-1.64\sqrt{\left(\frac{1}{a}+\frac{1}{b}+\frac{1}{c}+\frac{1}{d}\right)}}$	EB05≥2, N>0

ROR, reporting odds ratio; PRR, proportional reporting ratio; BCPNN, Bayesian confidence propagation neural network; MGPS, multi-item gamma Poisson shrinker; CI, confidence interval; χ^2^, chi-squared; IC, information component; IC025, the lower limit of the 95% two-sided CI of the IC; EBGM, empirical Bayesian geometric mean; EB05, the lower 90% one-sided CI of EBGM; N, number of cases.

### Statistical Analysis

A descriptive analysis was conducted to summarize the characteristic of TLS in patients receiving treatment with ICIs. For this, first, patients aged between 30 and 90 years were divided into 15-year groups (30–44 years, 45–59 years, 60–74 years, and 75–90 years), whereas those younger than 30 years or older than 90 years composed the other two groups. Second, the onset time was calculated and compared as the interval days between the start of ICI therapy and the onset of an adverse event; 10-day groups from 0 to 70 days after the onset of adverse events were established, and patients with the onset of adverse events more than 70 days after ICI administration were grouped together. Third, the outcomes considered were death, initial or prolonged hospitalization, life-threatening events, and other serious medical events according to the FAERS database. Additionally, the non-parametric Kruskal–Wallis test was used to compare the presence of TLS in different ICI subgroups. Data mining and statistical analysis were performed using SAS software 9.4 (SAS Inc., Cary, NC, United States).

## Results

All FDA-approved indications of the seven drugs are illustrated in Table 2. Pembrolizumab had 19 indications, covering eight main tissues or systems in the context of both hematology and solid oncology. The combined therapy of ipilimumab and nivolumab is marked with an a.

**TABLE 2:** Summary of immune checkpoint inhibitor indications.

	Tissue or system	Indications	Ipilimumab	Nivolumab	Pembrolizumab	Cemiplimab	Atezolizumab	Avelumab	Durvalumab
1	Hematopoietic and lymphoid	Classical Hodgkin lymphoma	-	6	5	-	-	-	-
		Primary mediastinal large B-cell lymphoma	-	-	6	-	-	-	-
2	Lung and thymus	Non–small-cell lung cancer	5^a^	2^a^	2	-	2	-	2
		Small-cell lung cancer	-	3	3	-	4	-	3
		Malignant pleural mesothelioma	6^a^	4^a^	-	-	-	-	-
3	Skin	Melanoma	1	1^a^	1	-	6	-	-
		Merkel cell carcinoma	-	-	14	-	-	1	-
		Cutaneous squamous cell carcinoma	-	-	18	1	-	-	-
4	Genitourinary	Renal cell carcinoma	2^a^	5^a^	15	-	-	3	
		Urothelial carcinoma	-	8	7	-	1	2	1
5	Gastroenteric and hepatic	Esophageal squamous cell carcinoma	-	11	11	-	-	-	-
		MSI-H or dMMR colorectal cancer	3^a^	9^a^	9	-	-	-	-
		Gastric cancer	-	-	10	-	-	-	-
		Hepatocellular carcinoma	4^a^	10^a^	13		5		
6	Head and neck	Squamous cell carcinoma	-	7	4	-	-	-	-
7	Uterine	Cervical cancer	-	-	12	-	-	-	-
		Endometrial carcinoma	-	-	16	-	-	-	-
8	Breast	Triple-negative breast cancer	-	-	19		3		
9	Non-specific	MSI-H or dMMR cancer	-	-	8	-	-	-	-
		Tumor mutational burden-high cancer	-	-	17	-	-	-	-

MSI-H or dMMR, microsatellite instability-high or mismatch repair deficient;

a represents the combined therapy of ipilimumab and nivolumab; -, not covered.

We identified 164 TLS cases among individuals receiving treatment with ICIs in the FAERS database, covering 5,709 individual TLS cases and 88,620 adverse events in individuals receiving treatment with ICIs reported between the first quarter of 2004 and the fourth quarter of 2020. The demographic and clinical profiles of the sample population are summarized in Table 3.

**TABLE 3:** Demographic and Clinical Characteristics of TLS in patients receiving treatment with ICIs.

	Anti-CTLA-4	Anti-(PD-1)/(PD-L1)	Anti-(CTLA-4+PD-1)	N (%)
Reporting year (N = 164)				
Before 2015	8	0	1	9 (5.49)
2015	2	10	1	13 (7.93)
2016	1	7	1	9 (5.49)
2017	1	15	1	17 (10.37)
2018	0	20	6	26 (15.85)
2019	0	19	9	28 (17.07)
2020	2	42	18	62 (37.80)
Age, years (N = 164)				
<18	0	0	1	1 (0.61)
30–44	2	8	4	14 (8.54)
45–59	1	16	13	30 (18.29)
60–74	9	48	8	65 (39.63)
75–90	2	15	6	23 (14.02)
Unknown	0	26	5	31 (18.90)
Sex (N = 164)				
Female	5	30	7	42 (25.61)
Male	9	69	27	105 (64.02)
Unknown	0	14	3	17 (10.37)
Reporter (N = 164)				
Physician	3	44	19	66 (40.24)
Pharmacist	2	5	4	11 (6.71)
Other health professional	4	21	7	32 (19.51)
Consumer	4	27	3	34 (20.73)
Unknown	1	16	4	21 (12.80)
Region (N = 164)				
Asia	4	55	21	80 (48.78)
Europe	5	22	3	30 (18.29)
North America	4	32	13	49 (29.88)
Oceania	0	2	0	2 (1.22)
South America	1	2	0	3 (1.83)
Country (n = 164)				
Argentina	0	2	0	2 (1.22)
Australia	0	2	0	2 (1.22)
Austria	0	1	0	1 (0.61)
Belgium	0	2	0	2 (1.22)
Canada	0	2	1	3 (1.83)
China	0	7	1	8 (4.88)
France	1	7	1	9 (5.49)
Germany	2	5	2	9 (5.49)
Greece	0	1	0	1 (0.61)
Israel	0	1	0	1 (0.61)
Japan	3	44	20	67 (40.85)
Mexico	0	0	1	1 (0.61)
The Philippines	0	1	0	1 (0.61)
Poland	0	1	0	1 (0.61)
Portugal	1	0	0	1 (0.61)
Spain	0	3	0	3 (1.83)
Thailand	0	2	0	2 (1.22)
Turkey	1	0	0	1 (0.61)
Ukraine	0	1	0	1 (0.61)
The United Kingdom	1	1	0	2 (1.22)
The United States	4	29	11	45 (26.83)
Venezuela	1	0	0	1 (0.61)
Indication (N = 162)				
Hematopoietic and lymphoid tissues	1	12	0	13 (8.02)
Lung and thymus	0	39	1	40 (24.69)
Skin	13	9	13	35 (21.60)
Genitourinary	0	12	11	23 (14.20)
Gastroenterologic and hepatic	0	11	1	12 (7.41)
Unknown	0	6	3	9 (5.56)
Head and neck	0	6	1	7 (4.32)
Soft tissue	0	2	3	5 (3.09)
Gastrointestinal and hepatocellular	0	3	1	4 (2.47)
Uterine	0	5	0	5 (3.09)
Unspecific	0	3	1	4 (2.47)
Central nervous system	0	1	2	3 (1.85)
Breast	0	1	0	1 (0.62)
Central nervous system	0	1	0	1 (0.62)
Onset time (days) (*N* = 81)				
0–10	5	28	7	40 (49.38)
11–20	1	4	4	9 (11.11)
21–30	0	4	4	8 (9.88)
31–40	0	4	2	6 (7.41)
41–50	1	0	2	3 (3.70)
51–60	0	5	2	7 (8.64)
61–70	0	2	1	3 (3.70)
>70	1	4	0	5 (6.17)
Outcome (*N* = 163)				
Death	13	47	11	71 (43.56)
Hospitalization – initial or prolonged	1	30	16	47 (28.83)
Life-threatening	0	6	2	8 (4.91)
Other serious (important medical event)	0	29	8	37 (22.70)

CTLA-4, cytotoxic T lymphocyte antigen-4; PD-1, programmed death receptor-1; PD-L1, programmed cell death 1 ligand 1; N (%), number of cases (percentage).

From the sample population, anti-CTLA-4, anti-(PD-1)/(PD-L1), and anti-(CTLA-4+PD-1) therapies were administered to 14, 113, and 37 cases, respectively. In the anti-(PD-1)/(PD-L1) group, nivolumab monotherapy was used in most cases (*n* = 55), followed by pembrolizumab (*n* = 34), atezolizumab (*n* = 17), avelumab (*n* = 3), durvalumab (*n* = 2), and cemiplimab (*n* = 2).

Annually, the number of ICI-induced TLS cases has increased gradually, with the most cases reported in 2020 (*n* = 62). Most cases involved individuals aged 60–74 years (*n* = 65). Male individuals (*n* = 105) were more vulnerable than female individuals (*n* = 42), with a mean age of 62.16 ± 16.30 years in male individuals and 62.15 ± 12.72 years in female individuals. Most of the reports indicated that physicians oversaw patient management (*n* = 66). Most cases were from Asia (*n* = 80) in terms of region and Japan (*n* = 67) in terms of country. TLS was reported in 149 cases of solid malignancies and 13 hematologic neoplasms. The top three malignancies were all solid tumors, including lung and thymus cancer (*n* = 40), melanoma (*n* = 35), and renal cell and urothelial carcinoma (*n* = 23).

After excluding records with incorrect entries, 81 cases with TLS onset time were analyzed. The median onset time of TLS in patients on anti-CTLA-4 therapy was 6 (interquartile range [IQR]: 2–39.5)* *days. The median onset time associated with anti-(PD-1)/(PD-L1) and anti-(CTLA-4 + PD-1) therapies was 9 (IQR: 2–40) and 20 (IQR: 7.5–37.75) days, respectively. There was no significant difference in the onset time among the three groups. Most cases had an onset time of 0–10 days (*n* = 40)—anti-CTLA-4 (*n* = 5), anti-(PD-1)/(PD-L1) (*n* = 28), and anti-(CTLA-4 + PD-1) (*n* = 7) cases.

In total, patient outcomes included death (*n* = 71), initial or prolonged hospitalization (*n* = 47), life-threatening events (*n* = 8), and other serious medical events (*n* = 37). The distribution of mortality among the three groups was significantly different (χ^2^ = 18.655, *p* < 0.001). Among these groups, the fatality rate of patients in the anti-CTLA-4 (92.9%) group was significantly higher than that in the anti-(PD-1)/(PD-L1) group (42.0%), (χ^2^ = 15.928, *p* < 0.001) and the anti-(CTLA-4 + PD-1) group (29.7%) (χ^2^ = 14.823, *p* < 0.001).

As shown in Table 4, based on the criteria for signal detection in each of the four algorithms, anti-CTLA-4, anti-(PD-1)/(PD-L1), and anti-(CTLA-4 + PD-1) therapies were identified. The signal values of combined therapy were higher than those of monotherapies, with an ROR of 6.80 (95% CI: 4.92–9.40), a PRR of 6.78 (χ^2^ = 181.27), an IC of 2.75 (IC025 = 1.99), and an EBGM of 6.74 (EB05 = 5.14).

**TABLE 4:** Detected signals of TLS related to ICIs by four algorithms.

Regimen	N	ROR (95%two-sided CI)	PRR (χ^2^)	IC (IC025)	EBGM (EB05)
Anti-CTLA-4	14	3.33 (1.97–5.63)	3.33 (22.75)	1.73 (1.02)	3.32 (2.14)
Anti-(PD-1)/(PD-L1)	113	3.92 (3.26–4.73)	3.92 (240.96)	1.95 (1.62)	3.86 (3.30)
Anti-(CTLA-4+PD-1)	37	6.80 (4.92–9.40)	6.78 (181.27)	2.75 (1.99)	6.74 (5.14)

CTLA-4, cytotoxic T lymphocyte antigen-4; PD-1, programmed death receptor-1; PD-L1, programmed cell death 1 ligand 1; N, number of cases; ROR, reporting odds ratio; CI, confidence interval; PRR, proportional reporting ratio; χ2, chi-squared; IC, information component; IC025, the lower limit of the 95% two-sided CI of the IC; EBGM, empirical Bayesian geometric mean; EB05, the lower 90% one-sided CI of EBGM.

## Discussion

To the best of our knowledge, this is the first study to comprehensively evaluate TLS in individuals receiving ICI treatment using data from real-world practice. The present frequentist analysis included the ROR and PRR, which have been utilized by the health authorities outside the United States, whereas the Bayesian analysis included the BCPNN and MGPS, which have been adopted by the WHO Uppsala Monitoring Centre and FAERS (Hauben et al., 2005). Compared with the performance using the receiver operating characteristic curve, area under the curve, and Youden’s index, which optimize the sensitivity and specificity levels, the Bayesian approach is superior to the frequentist approach in disproportionality analysis algorithms. The values of Youden’s sensitivity, specificity, and index of the four algorithms were as follows: BCPNN, 0.6549, 0.6847, and 0.3396; GPS, 0.6637, 0.6306, and 0.2943; ROR, 0.6549, 0.6577, and 0.3125; and PRR, 0.7080, 0.5856, and 0.2936, respectively (Pham et al., 2019). All four signal detection algorithms showed that TLS was significantly more frequent in individuals receiving treatment with ICIs than in those not treated with ICIs, whereas individuals receiving the combination therapy of ipilimumab and nivolumab had the highest reports of TLS.

Although the precise mechanism of how ICIs lead to cell lysis remains unclear, administrating humanized or fully human ICI monoclonal antibodies may activate an immune response leading to serious and even fatal complications in multiple organs. Among the cellular contents and metabolites released when neoplasm cells lyse, uric acid can induce nephrotoxicity by intrarenal crystallization, renal vasoconstriction, impaired autoregulation, decreased renal blood flow, oxidation, and inflammation; potassium can cause fatal cardiac arrhythmias and secondary hypocalcemia, leading to neuromuscular irritability, dysrhythmia, and seizure; calcium phosphate crystals may precipitate in various organs; and cytokines may cause systemic inflammatory response syndrome and multiorgan failure (Howard et al., 2011). Whether the onset of TLS is associated with the efficacy of ICI therapy or the spontaneous progression of various primary or metastasis neoplasm types remains unclear (Das and Johnson, 2019).

Patients can be classified as those having a high risk (HR), intermediate risk (IR), or low risk (LR) of developing TLS based on three sequential phases (Cairo et al., 2010). First, the diagnosis of laboratory TLS is defined based on any two or more serum values of uric acid (≥476 μmol/L or 25% increase from baseline), potassium (≥6.0 mmol/L or 25% increase from baseline), phosphate (≥2.1 mmol/L for children/≥1.45 mmol/L for adults or 25% increase from baseline), and calcium (≤1.75 mmol/L or 25% decrease from baseline) within 3 days before or 7 days after the initiation of chemotherapy. Second, the stratification of high-risk disease (HRD), intermediate-risk disease (IRD), and low-risk disease (LRD) according to tumor type, age of the patients, tumor stage, bulk disease, white blood cell (WBC) count, and lactate dehydrogenase (LDH) levels (Cairo and Bishop, 2004).

1) HRD: Burkitt lymphoma/leukemia; acute lymphoblastic leukemia (ALL) with WBC ≥ 100 × 10^9^/L; ALL with WBC < 100 × 10^9^/L and LDH ≥ 2× upper limit of normal (ULN); acute myeloid leukemia (AML) with WBC ≥ 100 × 10^9^/L; lymphoblastic lymphoma in an advanced stage; lymphoblastic lymphoma in an early stage with LDH ≥ 2× ULN; adult T-cell/diffuse large B-cell/peripheral T-cell/transformed/mantle cell lymphoma of children in stage III/IV with LDH ≥ 2× ULN; and adult T-cell/diffuse large B-cell/peripheral T-cell/transformed/mantle cell lymphoma of adult with LDH > ULN and bulky.
2) IRD: bulky, solid tumors, sensitive to chemotherapy including neuroblastomas, germ cell tumors, and small-cell lung cancer; chronic lymphocytic leukemia–targeted and/or biological therapies; ALL with WBC < 100 × 10^9^/L and LDH < 2× ULN; AML with WBC ≥ 25 × 10^9^/L and <100 × 10^9^/L; AML with WBC < 25 × 10^9^/L and LDH ≥ 2× ULN; lymphoblastic lymphoma in an early stage and LDH < 2× ULN; adult T-cell/diffuse large B-cell/peripheral T-cell/transformed/mantle cell lymphoma of children in stage III/IV and LDH < 2× ULN; adult T-cell/diffuse large B-cell/peripheral T-cell/transformed/mantle cell lymphoma of adult with LDH > ULN and non-bulky; and anaplastic/large-cell lymphoma of children in stage III/IV.
3) LRD: the remaining solid tumors, myeloma, acute leukemia, and lymphoma not mentioned in HRD and IRD.

The third step is the adjustment according to renal function and renal involvement.

1) HR: IRD with renal dysfunction and/or renal involvement; IRD with normal renal function and uric acid > ULN or phosphate > ULN or potassium > ULN.
2) IR: IRD with normal renal function; LRD of lymphoma and leukemia with renal dysfunction and/or renal involvement.
3) LR: LRD of lymphoma and leukemia with normal renal function; LRD of solid tumors and myeloma.

A preliminary search on PubMed did not reveal any report on TLS cases in the context of ICI phase 1 to 3 clinical trials. However, some metabolic disorders in the context of TLS including hyperkalemia and acute kidney injury were occasionally reported in association with pembrolizumab (Langer et al., 2016; Goldberg et al., 2020; Middleton et al., 2020), avelumab (Barlesi et al., 2018), and durvalumab uses (Massard et al., 2016). Currently, just eight case reports of individuals developing TLS while receiving treatment with ICIs or co-chemotherapy have been reported in the PubMed database (Masson Regnault et al., 2017; Fa’ak et al., 2019; Carrier et al., 2020; Magara et al., 2020; Narukawa et al., 2020; Shah et al., 2020; Sugimoto et al., 2020; Yen et al., 2020). All cases involved solid tumors—three cases of metastatic melanoma (Masson Regnault et al., 2017; Magara et al., 2020; Sugimoto et al., 2020), three cases of genitourinary malignancies (Fa’ak et al., 2019; Narukawa et al., 2020; Shah et al., 2020), one case of hepatocellular carcinoma (Yen et al., 2020), and one case of metastatic triple-negative breast cancer (Carrier et al., 2020). These patients were treated with atezolizumab, nivolumab, or ipilimumab monotherapy (Masson Regnault et al., 2017; Fa’ak et al., 2019; Sugimoto et al., 2020); combined therapy of nivolumab plus ipilimumab (Magara et al., 2020); co-chemotherapy with nanoparticle albumin-bound-paclitaxel and atezolizumab (Carrier et al., 2020); nivolumab plus sorafenib (Yen et al., 2020); pazopanib after nivolumab (Narukawa et al., 2020); or pembrolizumab plus axitinib (Shah et al., 2020). The age range of the patients was 37–79 years, with a mean age of 58.5 years. TLS onset was between 5 and 10 days after the initiation of therapy, with various clinical symptoms such as weakness, fatigue, nausea, vomiting, diarrhea, and abdominal pain. Five out of eight patients died within 3–22 days after hospital admission (Masson Regnault et al., 2017; Narukawa et al., 2020; Shah et al., 2020; Sugimoto et al., 2020; Yen et al., 2020) and one after being discharged for home hospice (Fa’ak et al., 2019).

The neoplasm-related intrinsic factors such as high proliferative rate, large malignancy burden, or high chemotherapy sensitivity might facilitate the onset of TLS (Cairo et al., 2010). The overall incidence of TLS in the real world is unknown; however, the reported TLS incidence is higher in patients with hematologic malignancies than in those with solid neoplasms (Gemici, 2006). In a study on prophylaxis with allopurinol, the overall incidence of TLS in 102 patients with high-grade non-Hodgkin lymphoma adults was 42% (Hande and Garrow, 1993). During treatment with aggressive intravenous hydration, urinary alkalinization, and allopurinol, the incidence of TLS among 218 children with Burkitt acute lymphoblastic leukemia was 16.1% (Wössmann et al., 2003). In a study that included 772 adult patients with AML, 130 patients developed TLS—laboratory TLS was 12% and clinical TLS was 5%; furthermore, clinical TLS was associated with a higher mortality rate (Montesinos et al., 2008). In patients with solid tumors, rare cases of TLS have been reported in 46 patients with metastatic germ cell tumors, 300 patients with small-cell lung cancer, and 90 patients with neuroblastomas, which were considered an IRD (Gemici, 2006). Our study revealed that TLS occurred in 13 cases of hematologic neoplasms and 149 cases of solid malignancies. This may be because ICI indications cover more solid tumors than hematologic malignancies, as shown in Table 2. The 13 reported cases of hematologic neoplasm consisted of lymphoma (*n* = 6), plasma cell myeloma (*n* = 2), lymphocytic leukemia (*n* = 2), acute myeloid leukemia (*n* = 1), acute lymphocytic leukemia (n = 1), and myelodysplastic syndrome (*n* = 1). These neoplasm types covered the HRD, IRD, and LRD; however, further analysis could not be conducted because data such as WBC count, LDH level, and renal function were not collected in the FAERS. It seems that the off-label usage of ICIs is common in patients with hematologic malignancies as only classical Hodgkin lymphoma and primary mediastinal large B-cell lymphoma were the FDA-approved indications. It was unexpected that TLS occurred more often in solid malignancies, especially those occurring in the lung and thymus, skin, genitourinary, gastroenteric, and hepatic systems. The four therapies, namely, monotherapy of ipilimumab, nivolumab, or pembrolizumab, and combined therapy of ipilimumab and nivolumab, were applied for the top three indications, non–small-cell lung cancer (*n* = 28), melanoma (*n* = 32), and renal cell carcinoma (*n* = 21), which could have led to the high reporting rates. With the growing application and efficacy of ICIs, awareness of the inherent factors that may predispose patients to potentially adverse effects is increasing. The increasing reports of previously rare cancer types imply that their incidence was previously underestimated.

The patient-related clinical features including pretreatment hyperuricemia or hyperphosphatemia, pre-existing nephropathy or exposure to nephrotoxins, oliguria and/or acidic urine, dehydration, blood volume depletion, elevated serum LDH, or inadequate hydration during treatment may cause the onset of TLS events (Hande and Garrow, 1993; Cairo et al., 2010; Howard et al., 2011). Our study revealed that age may be a risk factor as poor renal function, decreased heart and vascular stature, and complicated concomitant medication use might contribute to various clinical adverse events in elderly individuals. In a real-world pharmacovigilance study between renal adverse events and ICIs, atezolizumab showed a relatively stronger association with adverse events than other ICIs, and combined therapies, compared with monotherapies, strengthened the association. This study also reported sex difference. The average age of male and female patients was the same at around 62 years, although male patients seemed to be more susceptible and vulnerable to TLS than female patients in the three therapy groups. This may be associated with the high incidence of lung cancer, which is the leading cause of cancer deaths in men. In our study, the total TLS cases in male individuals were 2.5-fold higher than those in female individuals, and the number of male and female patients with lung and thymus cancers was 30 and 6, respectively.

Medication-induced TLS has been reported. TLS most often occurs after the initiation of cytotoxic therapy in patients with hematologic malignancies (Cairo et al., 2010). The use of single targeted anticancer drugs, including monoclonal antibodies, tyrosine kinase inhibitors, proteasome inhibitors, chimeric antigen receptor T cells, and proapoptotic agents, or combinations with conventional cytotoxic agents, could lead to an increase in the frequency and severity of TLS (Howard et al., 2016). In most cases, TLS was induced by chemotherapy in individuals with solid tumors (Gemici, 2006). The first FDA-approved drug was ipilimumab in 2011, whereas eight out of nine reported TLS cases involved patients receiving treatment with anti-CTLA-4 before 2015. Nivolumab and pembrolizumab were approved in 2014. From 2015 to 2017, 31 patients on anti-PD-1 regimen developed TLS. The 81 TLS cases in the anti-(PD-1)/(PD-L1) group between 2018 and 2020 accounted for 49% of the total cases; atezolizumab, avelumab and durvalumab, and cemiplimab entered the market in 2016, 2017, and 2018, respectively. The number of TLS cases receiving an anti-(CTLA-4 + PD-1) regimen increased from 2018 because the combined therapy was newly indicated for renal cell carcinoma, microsatellite instability-high (MSI-H), or mismatch repair deficient (dMMR) metastatic colorectal cancer in 2018 and hepatocellular carcinoma in 2020. It is noteworthy that the number of TLS cases has been increasing annually. The top two reporting countries were Japan (*n* = 67, 40.85%) and the United States (*n* = 44, 26.83%). As the country where the drug was originally developed and first approved for marketing, the annual reports from the United States did not exceed single digits between 2014 and 2020. In Japan, the first 16 TLS cases, involving patients on ipilimumab, nivolumab, a combination of ipilimumab and nivolumab, pembrolizumab, or atezolizumab, were reported to the FAERS before 2018. The increased number of annual reports in 2019 (*n* = 15) and 2020 (*n* = 36) implies the potential relationship between widespread usage, the active reporting behavior, and potential high morbidity. Therefore, it should be confirmed whether the populations in Asia and North America are particularly vulnerable. Among all 5,709 TLS events in FAERS, the medications associated with the high occurrence of TLS were venetoclax (*n* = 315), rituximab (*n* = 256), doxorubicin (*n* = 227), lenalidomide (*n* = 189), dexamethasone (*n* = 180), bortezomib (*n* = 156), carboplatin (*n* = 152), ibrutinib (*n* = 147), bevacizumab (*n* = 120), cisplatin (*n* = 112), prednisolone (*n* = 92), and cyclophosphamide (*n* = 73). Except for dexamethasone, carboplatin and prednisolone, TLS were labeled in “warnings and precautions” or “adverse drug reaction” sections on all FDA-approved inserts.

Our study emphasized the frequency, severity, and celerity of TLS events, with a noticeable occurrence of 2.87% in a total of 5,709 TLS events, a high ratio (43.56%) of fatality, and a quick onset (within 10 days of starting treatment). Identifying the ICIs related to TLS cases is important as they account for significant morbidity and mortality, unless recognized early and treated appropriately (Howard et al., 2011). ICI combination therapy showed the strongest correlation with TLS; however, the fatality rate in the anti-CTLA-4 monotherapy group was significantly higher than that in the combination therapy group. It is worth noting that 13 out of 14 patients in anti-CTLA-4 group died from 2011 to 2020. There were 12 cases with malignant melanoma, of which, five were metastatic melanoma and one was malignant melanoma of stage IV. The cancer type and stage might be crucial factors influencing the fatality rate.

Based on the rapid onset and effective prevention suggested by the expert panel (Cairo et al., 2010), it is essential to be aware of its causes, the physiologic consequences, and the predisposing risk factors, and to identify patients at high risk for TLS (Coiffier et al., 2008). The reported prophylaxis and treatment involving vigorous hydration to preserve renal function, close monitoring of electrolytes to maintain homeostasis, pretreatment with anti-hyperuricemia agents (allopurinol, febuxostat, or rasburicase), necessary dialysis, and further intensive care unit admission should be routinely performed in clinical practice (Alakel et al., 2017; Wagner and Arora, 2017). Two compassionate use trials reported rasburicase is safe and highly effective in the prophylaxis and treatment of malignancy-associated hyperuricemia in both children and adults (Bosly et al., 2003; Jeha et al., 2005). The issues of underreporting of ADR (Hazell and Shakir, 2006; Lopez-Gonzalez et al., 2009) and the attitude of health-care providers, including physicians (Herdeiro et al., 2005) and pharmacists (Herdeiro et al., 2006), are major factors in pharmacovigilance of SRSs. Therefore, not only health-care providers, who reported most of the cases in our study, but also the consumers themselves should be aware of TLS and the associated complications as the early recognition and treatment can mitigate the severity of TLS.

Although this study takes advantage of real-world research and novel data mining techniques, there are some limitations to consider. First, the FAERS does not contain data on all ICI treatment and TLS events in the world. Therefore, the incidence of suspected drugs and adverse events could not be calculated. Second, bias might exist based on missing or incorrect clinical information in the database. Third, confirmatory research should be carried out among individuals receiving combined therapy with ICIs and other anticancer medications. Finally, further studies are needed to explore the specific mechanism of how ICIs might induce TLS.

## Conclusion

We retrieved data from a large pharmacovigilance database and summarized several aspects regarding age, sex, region, country, indication, onset time, and outcome. Based on the early onset, non-specific symptoms, fulminant progression, and inferior outcomes identifying the association between ICIs and TLS are crucial. Oncologists and general clinicians should closely monitor high-risk patients with cancer who might develop TLS after the administration of therapeutic agents.

## Data Availability

The original contributions presented in the study are included in the article; further inquiries can be directed to the corresponding author.

## Glossary

ADR, adverse drug reaction; ALL, acute lymphoblastic leukemia; AML, acute myeloid leukemia; BCPNN, Bayesian confidence propagation neural network; CI, confidence interval; CTLA-4, cytotoxic T lymphocyte antigen-4; EBGM, empirical Bayesian geometric mean; FDA, Food and Drug Administration; FAERS, FDA Adverse Event Reporting System; HR, high risk; HRD, high-risk disease; ICIs, immune checkpoint inhibitors; ICH: international council for harmonization of technical requirements for pharmaceuticals for human use; IC, information component; IQR, interquartile range; IR, intermediate risk; IRD, intermediate-risk disease; LR, low risk; LRD, low-risk disease; LDH, lactate dehydrogenase; MGPS, multi-item gamma Poisson shrinker; PD-1, programmed death receptor-1; PD-L1, programmed cell death 1 ligand 1; PRR, proportional reporting ratio; PT, preferred term; ROR, reporting odds ratio; SMQ, standardized medical dictionary for regulatory activities query; SRS, spontaneous reporting system; TLS, tumor lysis syndrome; ULN: upper limit of normal; WBC, white blood cell; WHO, World Health Organization; χ2, chi-squared.
